# Retinal Organoids: Innovative Tools for Understanding Retinal Degeneration

**DOI:** 10.3390/ijms26073263

**Published:** 2025-04-01

**Authors:** Nadia Galindo-Cabello, Estefanía Caballano-Infantes, Gregorio Benites, Salvador Pastor-Idoate, Francisco J. Diaz-Corrales, Ricardo Usategui-Martín

**Affiliations:** 1 Department of Cell Biology, Genetics, Histology and Pharmacology, Faculty of Medicine, University of Valladolid, 47003 Valladolid, Spain; nadiaregina.galindo@uva.es; 2 Institute of Applied Ophthalmobiology (IOBA), University of Valladolid, 47011 Valladolid, Spain; benitesgregorio@gmail.com (G.B.); salvador.pastor@uva.es (S.P.-I.); 3 Department of Integrative Pathophysiology and Therapies, Andalusian Molecular Biology and Regenerative Medicine Centre (CABIMER), Junta de Andalucía, CSIC, Universidad de Sevilla, Universidad Pablo de Olavide, Avda. Américo Vespucio 24, 41092 Seville, Spain; francisco.diaz@cabimer.es; 4 Department of Ophthalmology, University Clinical Hospital of Valladolid, 47003 Valladolid, Spain

**Keywords:** retinal organoids, retinal degeneration, photoreceptors, stem cells

## Abstract

Retinal degenerative diseases (RDDs) comprise diverse genetic and phenotypic conditions that cause progressive retinal dysfunction and cell loss, leading to vision impairment or blindness. Most RDDs lack appropriate animal models for their study, which affects understanding their disease mechanisms and delays the progress of new treatment development. Recent advances in stem cell engineering, omics, and organoid technology are facilitating research into diseases for which there are no previously existing models. The development of retinal organoids produced from human stem cells has impacted the study of retinal development as well as the development of in vitro models of diseases, opening possibilities for applications in regenerative medicine, drug discovery, and precision medicine. In this review, we recapitulate research in the retinal organoid models for RDD, mentioning some of the main pathways underlying retinal neurodegeneration that can be studied in these new models, as well as their limitations and future challenges in this rapidly advancing field.

## 1. Introduction

Retinal degenerative diseases (RDDs), such as retinitis pigmentosa (RP) and age-related macular degeneration (AMD), are leading causes of blindness affecting millions of individuals worldwide. RP is the most common cause of adult hereditary blindness, while AMD is the most prevalent non-hereditary form of RDD, with its incidence increasing as populations age [1]. A hallmark of these conditions is the loss of photoreceptors and progressive retinal dysfunction. In RP, the primary degeneration occurs in rod and cone photoreceptors; however, in some cases, mutations can also affect the retinal pigment epithelium (RPE), as seen with *RPE65* or *MERTK* mutations [2,3]. Conversely, in AMD, the initial degeneration occurs in the RPE, which subsequently leads to the loss of cone photoreceptors in the central retina. In both diseases, the gradual decline in visual function ultimately results in blindness.

Within the broader spectrum of RDDs, inherited retinal diseases (IRDs) form a distinct subgroup characterized by heterogeneous genetic and clinical disorders affecting more than 5.5 million people worldwide [4]. RP is classified within IRDs, which have many inheritance patterns, including autosomal dominant, autosomal recessive, X-linked, and sporadic, with mutations in over 90 genes reported to date [4,5]. Currently, while there are limited treatments available for certain RDDs, most of these disorders lack effective therapeutic options, leaving a significant unmet medical need [6]. The lack of retinal cells’ regenerative capacity significantly contributes to retinal degeneration, exacerbating disease progression and challenging the development of new treatments [7,8].

The human retina is a light-sensitive tissue of the central nervous system located at the back of the ocular chamber. The retina comprises the RPE layer and the neural retina (NR), comprising three nuclear and two plexiform layers. The RPE, in its basal zone, rests on Bruch’s membrane, which separates it from the choroid, while in its apical zone, it interacts with the outer segments of the photoreceptors (OS), which are specialized in light detection. There are two types of photoreceptors: cones and rods, with their nuclei located at the outer nuclear layer (ONL). Next is the outer plexiform layer (OPL), where photoreceptor axons interact with horizontal and bipolar cells. Next is the inner nuclear layer (INL), composed of the nuclei of the bipolar, amacrine, horizontal, and Müller cells. The inner plexiform layer (IPL) is where bipolar and amacrine cells interact with retinal ganglion cells (RGCs). The nuclei of the RGCs form the RGC layer, while their axons form the optic nerve fiber layer [9,10].

As we can see, the retina’s structure is highly complex, with each layer playing a critical role in its function. In retinal degeneration, this intricate organization undergoes significant remodeling, as the gradual loss of neuronal cells leads to structural and functional alterations, which result in decreased vision. Multiple factors can trigger this process, including age, genetics, oxidative stress, inflammation, the aberrant proliferation of blood vessels, intraocular pressure variations, or metabolic disorders. These factors generally induce a set of signals at the molecular and cellular levels, activating cell death mechanisms and causing alterations in the morphology and functionality of retinal cells [11].

In this context, developing models that can recreate the processes of retinal neurodegeneration is of vital importance, as they enable studying at the molecular, physiological, and functional levels of these diseases, as well as accelerate the discovery of new therapeutic targets and the evaluation of new therapies [12]. Over the years, the understanding of retinal diseases has focused on studying animal models [13]. However, many human clinical phenotypes have not been fully simulated in these animal models [12]. In addition, using such models presents several limitations, including anatomical differences at the photoreceptor level, genetic variability between species, and ethical considerations [5,14]. On the other hand, the use of in vitro models for studying human diseases has been significantly influenced by the in vitro monolayer cell culture over the past decade. Nevertheless, the primary obstacle to faithfully representing biological processes in vivo has been the absence of culture complexity and tissue architecture [15].

A few years ago, advances in stem cell engineering and the development of omics and organoid technologies facilitated the generation of three-dimensional (3D) models from embryonic stem cells (ESCs) and induced pluripotent stem cells (iPSCs). Organoids are 3D multicellular structures created in vitro from ESCs, iPSCs, or neonatal or adult stem cells (ASCs) [15,16]. They comprise multiple kinds of cells and adhesion proteins, which give structural support. These in vitro models more accurately reproduce the cellular diversity and structural and functional complexity of real tissues, enabling better disease understanding at the molecular, cellular, and transcriptomic levels [15,17]. In the case of the retina, stem cell-derived retinal organoids enable a deeper understanding of the process of human retinogenesis and the generation of patient-specific in vitro models from their human-induced pluripotent stem cells (hiPSC) as part of personalized medicine [8,12,18].

Retinal organoids, often referred to as *miniature retinas*, exhibit a laminated, multilayered 3D structure composed of photoreceptors (cones and rods), horizontal cells, bipolar cells, amacrine cells, ganglion cells, and Müller cells, closely resembling the histoarchitecture of the native NR [18]. Although RPE cells may be present in retinal organoids, they do not grow as a monolayer in direct contact with photoreceptors. Moreover, retinal organoids have a functioning physiological ability to respond to light, like the in vivo retina [19]. Likewise, being derived from human cells (hESC or hiPSC), they can be carriers of gene variants, clarifying the causes of diseases in tissues and enhancing the comprehension of metabolic pathways and factors that affect disease progression [4]. The integration of organoid systems and the production of safe transplantable resources represent the future direction of this technology [12,17].

This review recapitulates research on developing retinal organoid models for neurodegenerative diseases and their limitations and future challenges. We comprehensively reviewed the literature through MEDLINE, PubMed, Web of Science, Scopus, and Embase electronic databases. Potentially relevant articles were sought by using the search terms in combination with Medical Subject Headings (MeSH) terms and text words: “organoids”, “retinal degeneration”, “photoreceptor cells”, “stem cells”, “pluripotent Stem Cells”, “retinal development”, and “therapy”. In addition, we examined the reference lists of the retrieved journals to find the more pertinent literature. The search was expanded with the MedLine ‘Related Articles’ option. No language restrictions were implemented. The abstracts of each publication were reviewed to confirm their relevance and significance.

## 2. Pioneering Research: From the Beginning of Retinal Organoids

Retinal organoids first appeared in 2011 when Eiraku et al. [20] detailed the creation of an autonomously forming optic cup structure derived from mouse embryonic stem cells (mESC). The study found that mESC aggregates could self-organize in three-dimensional cultures, forming hemispherical epithelial vesicles that differentiated into a rigid pigment epithelium in the proximal portion and stratified NR tissue in the distal portion, mimicking the optic cup’s development in vivo. A few months later, Meyer et al. [21] isolated and cultured optic vesicle-like (OV) structures from human embryonic stem cells (hESC) and hiPSC. These structures were recognized as multipotent retinal progenitors, capable of developing into photoreceptors and RPE. However, it was discovered that OV structures did not invaginate and create bilayered cups [21]. One year later, following Eiraku’s methodology, Nakano et al. [22] successfully created optical cups from hESCs. The main differences they identified compared hESCs to optic cups from mESCs are a larger diameter (550 µm versus 250–300 µm), the development time is longer (~24 days versus ~9 days), the NR is thicker and presents an apical convex curvature (120–150 µm versus 60–80 µm), and photoreceptor differentiation can be accelerated by Notch pathway inhibition. These findings reflected the variability in retinal development between species, suggesting that retinal self-organization and morphogenesis mechanisms may vary considerably between hESCs and mESCs [22]. Since then, different methods have been developed to create self-organized 3D retinal organoids from ESCs and iPSCs in vitro [19,23,24,25,26]. These methods focus on increasing the production reliability and identifying key factors that are responsible for a high-quality performance in large-scale production [7].

**Figure 1:**
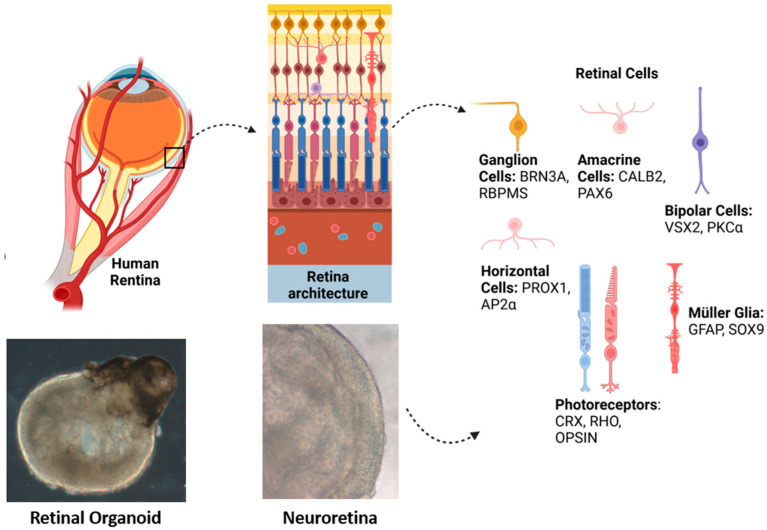
Architecture of the human retina and a retinal organoid. The different types of retinal cells and their main molecular markers detected in the retinal organoids are shown.

Figure 1 shows the correlation between the human retina and retinal organoids and the main molecular markers detected in retinal organoids. Regarding the expression of key markers in retinal organoids during their development, photoreceptors show the presence of CRX at Day 100 (D100), with an increase in the expression of RHO and OPSIN by Day 150 (D150) [27]. In bipolar cells, VSX2 expression is low at D100, while PKCα becomes visible at D150 [18]. Ganglion cells exhibit high levels of BRN3A at D100, with a decrease in RBPMS expression by D150 [28]. Amacrine cells maintain a consistent expression of CALB2 and PAX6 at both stages [18]. Müller glial cells show low GFAP expression at D100, but SOX9 is upregulated by D150 [29]. Finally, horizontal cells display moderate PROX1 expression at D100, with clear AP2α expression at D150 [18].

## 3. Generating Retinal Organoids: Growing and Differentiation Methods

There are three categories for generating retinal organoids which combine 2D and 3D cultures. The first category includes the classic approach described by Nakano et al. [22]. ESCs are fragmented into single cells and then reaggregated in 96-well plates with minimal cell adhesion. Then, on day 6, BMP4 is administered as a neuroepithelial inducer. On day 18, NR-like tissues are removed and cultivated for 6 days in the RPE-induction medium, followed by the retinal maturation medium. RGCs are first recognized around day 30, and photoreceptors show around day 130. The second category combines 2D and 3D cultures [24,30]. It starts with the culture of iPSCs at 70% confluence and the replacement of essential medium six without FGF2 and TGFb. On the second day, the N2 supplement is added. Subsequently, at 4 weeks, NR-like structures are selected and transferred to a suspension culture medium to start the 3D stage of long-term culture. The third category comprises the creation of embryoid bodies (EB) [23]. To create EBs, ESCs are fragmented into tiny clumps and grown in mTeSR1 media with Blebbistatin. EBs are subsequently moved to a neural induction medium, producing EB aggregates. On day 7, EBs are placed on matrigel-coated plates or flasks, where NR-like structures grow over the course of four weeks. The retina-like structures are then selected, dissected, and put in long-term orbital rotation.

Various molecules have been used for the generation of retinal organoids, both in the early induction phase and spheroid formation, as well as in retinal differentiation, maturation, and the stratification of organoids. The main signaling pathways modulated during this process include Wnt, Hedgehog, and FGF. A list of these molecules and their primary roles in retinal organoid development is provided in Table 1. The molecules have been grouped according to differentiation stages, but this classification does not imply that all of them must be used. In some cases, the same molecule may be beneficial in multiple stages. Currently, several optimized protocols exist, and selecting molecules from this list can serve as a guide when designing differentiation protocols.

**Table 1:** Molecules for retinal organoid generation at different developmental stages.

**Stage 1: Neuroectoderm Induction**	
**Molecule**	**Role**
Noggin [18]	Inhibits BMP signaling, promoting neuroectoderm specification.
SB431542 [31]	TGF-β pathway inhibitor, enhances neuronal differentiation.
LDN-193189 [31]	BMP inhibitor, promotes differentiation into neural progenitors.
BMP4 (Bone Morphogenetic Protein 4) [32]	Induces neural ectoderm formation and promotes early retinal differentiation.
**Stage 2: Retinal Specification**	
**Molecule**	**Role**
IGF-1 (Insulin-like Growth Factor 1) [32]	Promotes the differentiation of retinal progenitors.
bFGF (Basic Fibroblast Growth Factor, FGF2) [33]	Supports neural and early retinal progenitor proliferation, initiating differentiation toward retinal fate.
IWR-1 (Inhibitor of Wnt Response 1) [22]	Inhibits Wnt signaling, facilitating retinal cell differentiation and organized neuroretinal epithelium development.
Dkk-1 [34]	Wnt inhibitor, promotes differentiation into retinal progenitors.
Activin A [22,35]	Enhances spheroid formation by inducing the expression of early retinal development genes such as PAX6, supporting retinal progenitor proliferation.
**Stage 3: Optic Vesicle and Neuroretina Formation**	
**Molecule**	**Role**
Retinoic Acid (RA) [22,35]	Potent inducer of differentiation and retinal layer formation, promoting progenitor specification via CRX and PAX6 expression. However, it could delay maturation.
Sonic hedgehog agonist SAG [22,35]	Regulates retinal progenitor proliferation and retinal pigment epithelium (RPE) organization.
EGF (Epidermal Growth Factor) [36]	Supports proliferation and survival of retinal progenitor cells and it is useful to isolate and maintain Müller cells.
Wnt3a [22]	Prevents premature differentiation of retinal progenitors and maintains stem cell pluripotency.
CHIR99021 (GSK-3β inhibitor) [22]	Activates Wnt signaling, promoting early optic neuroepithelium growth and epithelial differentiation with MITF positive cells.
BMP4 [32]	Regulates RPE specification.
**Stage 4: Retinal Differentiation and Maturation**	
**Molecule**	**Role**
B27, non-essential amino acid solution (NEAA) and N2 supplements [33]	Essential for maintaining optimal stem cell growth and differentiation conditions, supporting the survival and structural organization of retinal organoids.
DAPT (Notch inhibitor) [22]	Inhibits Notch signaling, promoting retinal progenitor differentiation and precise stratification, mimicking human retina organization.
BDNF (Brain-Derived Neurotrophic Factor) and CNTF (Ciliary Neurotrophic Factor) [37]	Neurotrophic factors that support retinal cell survival and maturation, facilitating photoreceptor layer development and Müller glia differentiation.
SU5402 [38]	FGF inhibitor, promotes photoreceptor maturation and RPE differentiation.
**Stage 5: Advanced Maturation and Functional Photoreceptors**	
**Molecule**	**Role**
Taurine [39]	Sulfonated amino acid critical for photoreceptor development, neuroprotection, and calcium homeostasis. Enhances neurotrophic factors’ effects and accelerates retinal organoid maturation and stratification.
9-cis-Retinal [40]	Opsin cofactor, essential for functional photoreceptor maturation.
DAPT (Notch inhibitor) [22]	Inhibits Notch signaling, promoting retinal progenitor differentiation and precise stratification, mimicking human retina organization.

**Figure 2:**
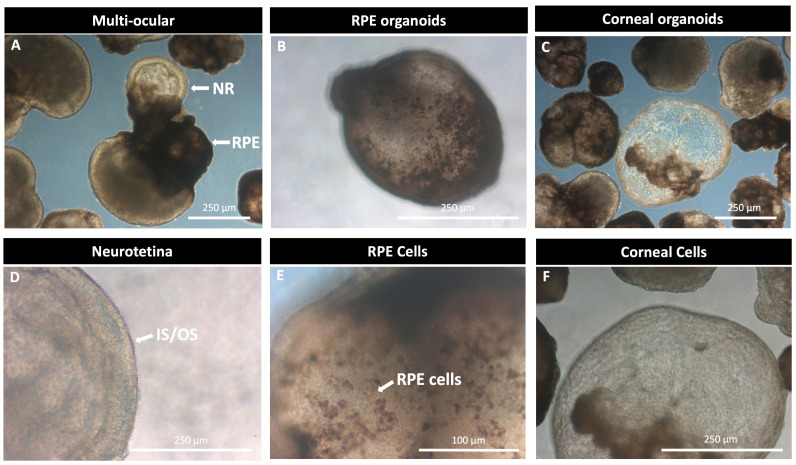
Multi-ocular differentiation protocol from human iPSCs-wt. This figure illustrates the heterogeneity of cultures following the basal differentiation protocol described by Cowan et al. [34] with modifications in our lab. In panel (**A**), a multi-ocular differentiation is shown from a 100-day differentiation system, where the retinal pigment epithelium (RPE), corneal zone, and neuroretina (NR) can be observed. Panel (**B**) displays a 3D structure corresponding to an RPE-specific organoid. Panel (**C**) highlights a central structure corresponding to a corneal organoid. In panel (**A**), a multiocular organoid is observed. Panel (**B**) shows pigmented hexagonal RPE cells. Panel (**C**) clearly displays cells similar to those found in the cornea. In panel (**D**), the differentiation of the neuroretina and the inner (IS) and outer segments (OS) of the photoreceptors is observed. In panel (**E**), hexagonal pigmented cells characteristic of the RPE are observed. In panel (**F**), epithelial cells resembling those of the cornea are observed.

Methods are continually being modified and optimized to produce specific populations of cells and organoids with higher levels of differentiation and maturation. Isla-Magrané et al. [41] demonstrate that allowing the self-organization of multiocular structures derived from hiPSCs could produce complex 3D multicellular ocular organoids that include the retina, cornea, retinal pigment epithelium, and stroma. The hiPSC-derived organoids in this study demonstrate potential applications in developmental biology research and disease models for the anterior and posterior segments of the eye [42]. Building on these findings, our research group has also observed diverse 3D structures that highlight the heterogeneity inherent in retinal cell differentiation (Figure 2). This variability suggests that cellular development is a highly intricate process, which may not be fully captured by traditional transcriptomic analyses. Consequently, single-cell studies and other advanced techniques are recommended to achieve a more comprehensive understanding of these processes.

## 4. Metabolic Changes in Retinal Organoids

Retinal organoids serve as valuable models for studying retinal development and disease, yet their metabolic environment differs significantly from in vivo conditions. The oxygen supply and metabolic demand are critical in shaping cellular function within these systems. Unlike the vascularized retina, organoids rely on passive diffusion for oxygen and nutrient exchange, which can lead to hypoxic regions, particularly in later developmental stages. Hypoxia-inducible factors (HIFs) become activated under these conditions, potentially influencing photoreceptor differentiation and survival. Additionally, metabolic shifts from oxidative phosphorylation to glycolysis may occur, resembling the Warburg effect observed in degenerative and proliferative retinal diseases. A recent study found that retinal organoids with a heterozygous RB1 mutation exhibited a metabolic shift from glycolysis to oxidative phosphorylation, leading to increased ATP production, highlighting the importance of metabolic regulation in these models [42]. Strategies such as dynamic oxygenation systems or metabolic supplementation (e.g., lactate and pyruvate) are being explored to optimize organoid maturation and functionality, bridging the gap between in vitro and in vivo retinal metabolism. Understanding these metabolic dynamics is crucial for improving the physiological relevance of retinal organoids as disease models and therapeutic platforms.

### 4.1. Oxygen Gradient in the Retina and Its Importance in Retinal Development and Function

Studies highlight that the retina exhibits a crucial oxygen gradient essential for its development and function. The inner retina exists in a hypoxic environment (~2% O_2_), whereas oxygen levels increase toward the choroid’s outer retina (~18% O_2_). This gradient plays a key role in maintaining the health of retinal ganglion cells and other retinal cell types [43].

### 4.2. Influence of Hypoxia on Retinal Progenitor and Ganglion Cells in Retinal Organoids

Research indicates that low-oxygen conditions promote the proliferation and maintain the pluripotency of human-induced pluripotent stem cell-derived retinal progenitor cells (hiPSC). However, hypoxia also influences ganglion cell differentiation, suggesting that oxygen regulation is critical for the proper development of retinal organoids [44].

### 4.3. Challenges in Oxygenation and Metabolism in Retinal Organoid Cultures

Retinal organoids cultured under static conditions may experience limitations in oxygen and nutrient delivery, affecting their growth and differentiation. Dynamic culture methods, such as rotating-wall vessel bioreactors, have shown improvements in oxygenation, thereby enhancing organoid development [45].

### 4.4. Key Metabolic Challenges in Retinal Organoids

- Oxygenation Limitations: Unlike the highly vascularized in vivo retina, retinal organoids depend on the passive diffusion of oxygen and nutrients. As they grow, they may develop hypoxic regions that affect cellular differentiation and photoreceptor viability.
- Impact on Cellular Maturation: Oxygen availability regulates key retinal processes, such as the differentiation of ganglion and photoreceptor cells. Low oxygen levels can activate HIFs, which may influence gene expression and cellular metabolism.
- Metabolic Differences from the Native Retina: In physiological conditions, photoreceptors consume large amounts of oxygen and depend on oxidative phosphorylation. If organoids fail to replicate this environment, they may have an altered function and may not serve as accurate disease models.
- Strategies to Improve Oxygenation: Novel approaches, such as metabolic supplementation, bioreactor-based culture systems, or vascularized organoid engineering, could enhance oxygenation and make these models more comparable to the native retina.

By addressing these metabolic challenges, researchers can improve the physiological relevance of retinal organoids and their utility in disease modeling and therapeutic development.

## 5. The Cellular Mechanisms of Neurodegeneration in the Retina and the Utilization of Retinal Organoids for Research

**Figure 3:**
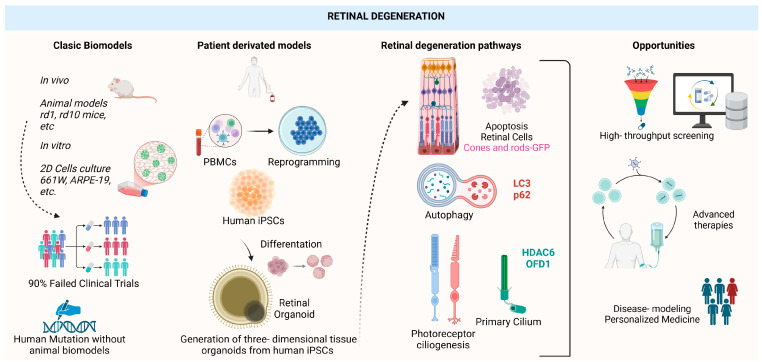
Generation of three-dimensional models based on the development of retinal organoids to study the mechanisms of retinal degeneration in various diseases. Initially, classical models were used to study these diseases, such as in vivo models like the rd10 mouse for RP research. It was also common to use 2D in vitro culture models with photoreceptor precursor cell lines, such as the murine 661W line or the human RPE line ARPE-19. However, the limitation of these basic experimental models is that they do not accurately mimic human diseases, and it has been observed that approximately 90% of clinical trials fail in the early stages. Additionally, another significant limitation is that certain human mutations causing retinal diseases occur in genes that are not conserved in murine models. Therefore, the generation of 3D in vitro models derived from patient iPSCs represents a significant breakthrough, enabling the precise and personalized study of various retinal pathologies. These models allow for the investigation of key pathways involved in retinal neurodegeneration, such as photoreceptor apoptosis, autophagy (LC-3 and p62), and ciliogenesis (HDAC6 and OFD1). Furthermore, these models serve as a valuable tool for high-throughput drug discovery, the development of advanced gene-editing therapies, and the design of personalized medicine.

Neurodegeneration is a complex process that involves various cellular mechanisms, including apoptosis, autophagy, and alterations in ciliogenesis, among others [41]. As we have mentioned before, the loss of photoreceptors and other retinal neurons is a key event in degenerative retinal diseases. In this review, we explore how these three major pathways—apoptosis, autophagy, and ciliogenesis defects—can be studied using retinal organoids. While these are relevant mechanisms implicated in retinal degeneration, many other pathways could also be investigated using these models. Several studies have identified disruptions in these pathways that are linked to the pathophysiology of hereditary retinal degenerative diseases. Understanding these mechanisms in organoid-based models can provide valuable insights into potential biomarkers and facilitate drug screening (Figure 3), as these models closely mimic the physiological conditions of the human retina [42,43].

### 5.1. Apoptosis

Apoptosis is one of the mechanisms of cell loss described in degenerative retinal diseases [11,46]. Studies have shown that oxidative stress, genetic mutations, and environmental damage can trigger photoreceptor apoptosis. Retinal organoids have made it possible to observe how photoreceptors respond to different apoptotic stimuli and how certain therapies can inhibit this process [47]. The evaluation of apoptosis in retinal organoids can be performed using several techniques, such as flow cytometry, immunofluorescence, and others [48]. Flow cytometry is used to analyze GFP+ rod-type photoreceptors, allowing the quantification of apoptotic cells and overall cell viability at different stages of development.

On the other hand, rhodopsin is used as a marker for rods, and opsin is used to analyze cones by immunofluorescence staining. Both methodologies allow the evaluation of wild-type (WT) retinal organoids and those derived from iPSCs with different mutations. This approach allows the comparison of untreated samples and organoids treated with non-toxic compounds, providing information on how these interventions affect apoptotic pathways [48]. Su and collaborators [49] developed retinal organoids and RPE cells from the hiPSC of an RP patient (*USH2A* mutation). They demonstrated that the *USH2A* gene mutation induces the apoptosis of hiPSCs and retinal organoids. The expression of anti-apoptotic protein BCL2 was observed to decrease, whereas pro-apoptotic protein BAX was increased in the retinal organoid with *USH2A* mutation. Furthermore, a higher percentage of TUNEL-positive cells (apoptotic cells) was identified in retinal organoids on day 80 of the culture, suggesting that a mutation in *USH2A* induces a process of apoptosis in these models. A deficiency of the extracellular matrix (ECM) components, such as laminin and collagen IV, was also observed.

RP11-RO-derived photoreceptors showed notable morphological defects, featuring a 1.5-fold rise in apoptotic nuclei compared to controls. The presence of stress vacuoles, a hallmark of adaptive cellular survival, accompanied this. Additionally, RP11 organoids demonstrated a reduced spiking rate of retinal ganglion cells (RGCs) in response to the neurotransmitter GABA, as assessed using multi-electrode arrays (MEAs). The measurement of RGC activity serves as a key indicator of neural connectivity among retinal cells within the organoids. The observed reduction in RGC activity suggests the impaired formation and functionality of retinal networks in the RP11 retinal organoids [50].

### 5.2. Autophagy

Autophagy is a mechanism of cellular self-degradation and recycling of damaged intracellular components and misfolded proteins, playing an essential role in cell survival, differentiation, development, and homeostasis [51,52]. In the retina, autophagy dysfunction has been linked to the accumulation of toxic proteins and cell death [52,53,54]. Key aspects of autophagy include the formation of autophagosomes and their degradation within lysosomes, all regulated by the mTOR pathway [53]. Retinal organoids facilitate the study of the influence of how alterations in autophagic mechanisms or their dysfunction contribute to cell degeneration, focusing on the evaluation of autophagosome formation in retinal organoids subjected to metabolic stress [55] or the accumulation of waste or misfolded proteins [56]. Another cytoplasmic protein involved in autophagy is p62 (encoded by the Sequestosome 1 gene (SQSTM1)), which binds to ubiquitinated or aggregated proteins, forming a complex that facilitates the formation of autophagosomes for their degradation [57,58]. In retinal organoids derived from iPSCs of patients with Leber congenital amaurosis type 10 (LCA10), significantly lower levels of p62 were detected compared to the controls.

Furthermore, another key regulator of autophagy, LC-3B, exhibited significantly higher levels of LC3-II in *LCA10* patient-derived organoids (mutations in the cilia-centrosomal *CEP290* gene), evidencing autophagosome accumulation and defects in the autophagy. The comparable ratio of LC3-II/LC3-I between the control versus patient organoids suggested that autophagosome formation rates were similar, implying that the increased levels of LC3-II in patient organoids might be due to autophagosome accumulation [48]. The development of retinal organoids from patients with mutations associated with retinal degeneration could allow for studying autophagy pathways, providing a deeper understanding of their role in the pathology and identifying potential targets for future therapies.

### 5.3. Ciliogenesis and Ciliopathies in the Retina

Primary cilia are cellular structures that operate as sensory organelles and facilitate cellular contact. In the retina, photoreceptor cells produce phototransduction-specific proteins and feature immotile cilia that help with signal transduction. Several ciliopathies result from defects in cilia production, transport, or function. These issues may manifest as sensory anomalies or as part of a larger syndromic disorder that affects several organs. Common symptoms include impaired kidney development, brain abnormalities, and retinal degeneration, such as RP [59]. The formation and maintenance of the primary cilium play a fundamental role in phototransduction and are one of the crucial elements of ciliogenesis. Relevant research has examined the role of ciliogenesis-related genes in retinal organoids, which develop into functioning photoreceptor structures that include cilia, offering insights into the molecular processes causing these illnesses.

Some studies have reported a link between autophagy and primary cilium biogenesis [60,61,62]. Therefore, examining the expression of key regulators involved in ciliary transport, assembly, and disassembly is crucial for understanding the role of ciliogenesis in the pathogenesis of retinal degeneration, such as in *CEP290* mutations associated with LCA [48]. CEP290-related retinal ciliopathies were associated with an increased expression of OFD1 (Oral-Facial-Digital Syndrome 1). OFD1 is related to the initiation of ciliogenesis, but it is usually degraded by autophagy to stimulate cilia development [61]. However, patient-derived organoids had greater levels of OFD1 than controls despite enhanced autophagy activity in the latter [48]. This divergence might be attributed to differences in the autophagic adapter machinery responsible for cargo recognition.

Nonetheless, lowering OFD1 levels in patient organoids is expected to facilitate the initiation of cilia biogenesis. Another important regulator, histone deacetylase 6 (HDAC6), which deacetylates microtubules and destabilizes the primary cilium for disassembly [63,64], was significantly elevated in LCA patient organoids compared to controls. In this context, HDAC6 could be a promising target for intervention, as reserpine has been shown to positively affect cilia biogenesis in LCA patient organoids by reducing HDAC6 levels. Consistent with this hypothesis, the treatment of patient organoids with Tubastatin A, a selective HDAC6 inhibitor, led to improved rod photoreceptor development, evidenced by a higher number of RHO+ cells [48]. Tubastatin A treatment also enhanced the polarity of rhodopsin and S-opsin, likely by stabilizing microtubules and improving intracellular trafficking. The quantification of the rhodopsin fluorescence intensity showed a significant increase in rhodopsin expression in Tubastatin A-treated organoids, suggesting that HDAC6 inhibition positively impacts rod photoreceptor development [48]. These findings indicate that ciliogenesis plays a critical role in maintaining the stability of rods and cones. Therefore, the further analysis of 3D patient-derived organoids could help design genetic or pharmacological strategies to treat ciliopathies more effectively. Retinal organoids provide a unique window for exploring the cellular mechanisms underlying neurodegeneration. Investigating how apoptosis, autophagy, and ciliogenesis are altered in these models will enable the development of more effective therapeutic strategies for treating degenerative retinal diseases.

Furthermore, a comparable phenotype was observed in RP11 retinal organoids and the RP11 retinal pigment epithelium (RP11-RPE) due to *PRPF31* mutations, where cilia appeared abnormal, exhibiting a bulbous structure and disorganized microtubules. The link between this phenotype and *PRPF31* mutations was validated using siRNA-mediated knockdown in a human RPE cell line, successfully replicating the characteristic disease features [65].

## 6. Disease Modeling with Retinal Organoids

RDDs comprise a wide range of conditions that can arise from genetic alterations, aging, or injury, ultimately resulting in photoreceptor loss, remodeling, and the degeneration of the entire retina [66]. Currently, retinal organoids serve as a tool that facilitates the study of these retinal neurodegenerative disorders, as they provide a 3D perspective of their structure and facilitate the study of the influence of genes and environmental factors on the health and survival of their cells [17]. Likewise, retinal organoids are widely used as models for the study of retinal neurodegeneration, from the processes of retinal development to the mechanisms and pathophysiology of diseases, disease detection, and new forms of treatment [67].

IRDs comprise the main object of study with retinal organoids. This includes several forms of RP, such as X-linked RP, Non-syndromic RP, and Late-onset RP; additionally, conditions like Leber congenital amaurosis (LCA), Stargardt disease, Usher syndrome, Joubert syndrome and Related Disorders (JSRD), and X-linked Juvenile Retinal Retinoschisis (XLRS) have also been investigated using this approach [4,6,16,67,68,69,70].

The main objective of the use of organoids in the study of IRDs is to analyze gene mutations associated with these pathologies. In this sense, it begins with the analysis of development using hiPSC-induced retinal organoids and RPE monolayers, retinogenesis (retinal organoids that can mimic embryonic development), the cell structure (e.g., cilia), spatiotemporal distribution, and the dead which are undercover, as well as the expression of differentiation markers, retinal disease genes, and mRNA alternative splicing [4,6,67,69,71,72]. Table 2 summarizes the principal retinal organoid models for the study of the IRDs’ pathophysiology. Retinitis Pigmentosa, or cone–rod dystrophy (CRD), is the main hereditary group of disorders studied using retinal organoids. Key genes like *CRB1*, *PRPF31*, and *RPGR* are the primary focus of study [66,68,73]. LCA is another condition widely analyzed in multiple studies, which focuses on mutations in the genes *CRB1* (photoreceptor ciliary transport) and *RPE65* (retinoid cycle) [4,74]. Stargardt disease, considered the most common juvenile form of macular degeneration, exhibits its principal issue as a mutated *ABCA4* gene, which has been the subject of study [56,75]. Usher syndrome, with type one of four being the most severe, has multiple mutations and genes associated with *USH2A, MYO7A,* and *USH1B*, which are the most commonly studied using retinal organoids [69,76]. In contrast, age-related macular degeneration, as an acquired retinal disease, has sparse existing literature on the application of retinal organoid technology to it; however, current study concepts are attempting to develop the transplantation of RPE sheets over degenerated retinas, with many trials ongoing [72].

Once the genes and processes involved are identified, studies focus on developing targeted treatments [95]. Two innovative techniques that have shown great promise are CRISPR-Cas9 editing, which allows the production of specific cell lines or the correction of genomic alterations, and AAV-mediated gene repair, which involves directly modifying the underlying genetic defect using viral delivery systems. Since the successful edition of the c.2991+1655A>G mutation of the ciliary gene *CEP290*, by Maeder et al., combining these methods has garnered significant interest [69].

Consequently, these approaches have demonstrated promising results in addressing mutations in several other genes (Table 3), such as *RPGR*, *CRB1*, *CRX*, *RP2*, *RS1*, *AIPL1*, and *NPHP5*, in the author’s style to edit certain cell lines generating retinal organoids that mimic certain diseases, proceeding to implement different AAV vectors to correct the mutation, like Sladen et al. [96]. Others like da Costa et al. [97] take patient-derived iPSCs and apply gene repair to generate corrected retinal organoids. Parallel to this, many other options have been developed, such as protein translocation therapies, biologics (e.g., RNA oligonucleotides), and small non-biological molecules (e.g., Eupatilin).

Ultimately, these efforts converge on transplanting healthy or genetically modified retinal organoids into animals or human patients for assessing survival, hoping to restore their vision and improve their quality of life [6,16,67,68,71,72,98]. Despite human transplantation being the latest form of therapy developed, no current large trials have started and many challenges remain with it. The four main modalities have already been described in animals (mostly mice, rats, squirrels, and monkeys), transplanting retinal single cells, organoid sheets, RPE, and co-grafts of RPE and organoids, each one with different rates of integration and light vision restoration [99,100]. Currently, great advances have been reached regarding the survival of transplanted retinal organoids on animal hosts, visual improvement/recovery, inner segment development from the donor photoreceptor, and host–graft synapses. Nevertheless, the visual recovery is small, the histologic evaluation of the outcomes interrupts the long-term follow-up and is needed to demonstrate significant changes in the host retinas, and novel imaging techniques for phenotypic evaluation are still in development [99,100,101]. Few human transplantation experiments have been performed which present promising results regarding the survival of the transplanted retinal organoids; however, their complexity is limited to open-label-limited cases with no control groups and few patients, they are taking the step of assessing safety, and a long-term visual acuity improvement has not been observed, though some functional improvements were transiently noted (Table 3) [102,103].

**Table 3:** Models for the treatment of IRDs and acquired retinal degeneration diseases with retinal organoids [4,6,16,67,68,69,71,72,102].

Treatment	Disease	Treatment Studies	
CRISPR-Cas9-mediated correction	RP	- Production of hiPSCs derived from a CRB1 patient, to correct the c.2480G>T, p.(Gly827Val) CRB1 mutation (da Costa et al., 2023).	[97]
		- Treatment of defects (morphology issues like cilium modification, cellular localization, gene expression profiling, and electrophysiological activity) (Deng et al., 2018).	[104]
		- *RPGR* mutations exert a rescue effect on photoreceptors (Deng et al., 2018).	
	LCA	- Correction of a nonsense variant associated with LCA type 5 (Afanasyeva et al., 2023).	[105]
		- LCA type 7 *CRX* gene editing on naïve pigmented retinas (Chirco et al., 2021).	[83]
AAV-mediated gene repair	RP	- AAV-*RPGR* gene repair applied to retinal organoids created through CRISPR/Cas9-mediated *RPGR* knock-out (Sladen et al., 2024).	[96]
		- AAV-mediated CRB1 gene augmentation therapy to restore cellular features and transcriptional processes (Boon et al., 2023).	[85]
	LCA	- Ciliary gene *NPHP5* in LCA using AAV2 (Kruczek et al., 2022).	[106]
	X-linked RP	- *RP2*-null X-linked RP model generated from CRISPR/Cas9-edited iPSC lines utilized to evaluate *RP2* gene therapy delivered via AAV5 (Lane et al., 2020).	[89]
		- Gene therapy targeting ciliopathies in a patient-derived model with *RPGR* mutations was explored using AAV2-7m8 to deliver a shortened version of RPGRORF15 (West et al., 2022).	[107]
	XLRS	- Augmentation of the *RS1* gene to prove developmental delay (Liang et al., 2023).	[71]
		- AIPL1 therapy to restore key molecular functions, reducing elevated cGMP (Sai et al., 2024).	[108]
Protein trans-splicing	-	- Reconstitute full-length *CEP290* and *ABCA4* in animal and retinal organoid models, with intein-mediated recombination (Tornabene et al., 2019).	[109]
QR-110 (RNA oligonucleotide)	LCA	- Defective pigmented retina ciliation correction on LCA 10 gene *CEP20* (Dulla et al., 2018).	[79]
Eupatilin	LCA	- Flavonoid showed to improve cilium formation (Corral-Serrano et al., 2023).	[110]
Reserpine	LCA	- Found to maintain photoreceptor survival on rd16 mouse models of LCA10, which showed *CEP290* null mutations induced by CRISP/Cas9 techniques (Chen et al., 2023).	[48]
AA147 (Small molecule ATF6 agonist)	Achromatopsia	- Correction of non-formation of cone structures and opsins (Kroeger et al., 2021).	[111]
Stem-cell transplantation using retinal organoids	-	- Cones from hiPSCs-derived retinal organoids transplantation into healthy and retina-damaged squirrels for survival assessment (Yu et al., 2024).	[99]
	RP	- Human photoreceptors from hESC-derived retinal organoids transplantation into blind rats for survival and visual improvement assessment (Lin et al., 2024).	[100]
	RP	- Human photoreceptors from hiPSCs-derived retinal organoids transplantation into mice with cone degeneration, using an rd1 mouse model (Ribeiro et al., 2021).	[112]
Direct transplantation of cultured retinal organoids tissue as a retinal sheet into animals and human retinas	RP	- Allogeneic hiPSCs-derived retinal organoids, from which dissected retinal tissue was transplanted into the eyes of patients with RP (Zhang et al., 2024 and Hirami et al., 2023).	[102,103]
	-	- Co-grafts made of human retinal photoreceptor progenitor sheets and RPE cells on a membrane, over immunodeficient rats with advanced stages of neurodegeneration (Biju et al., 2021).	[113]
	Age-related macular degeneration	- Retinal organoids transplantation clinical trials and concepts of study (Maeda et al., 2022).	[72]

CRISPR: Clustered Regularly Interspaced Short Palindromic Repeats; Cas9: CRISPR-associated protein 9; AAV: Recombinant Adeno-Associated Virus; AAV5: AAV serotype 5; AAV2: AAV serotype 2; AAV2-7m8: AAV serotype 2-7m8; RP: Retinitis Pigmentosa; *RPGRORF15*: isoform of the RPGR gene; RS1: Retinoschisin 1; *LCA*: Leber Congenital Amaurosis; *NPHP5*: Nephrocystin-5; *CRX*: Cone–rod homeobox; *CRB1*: Crumbs homolog 1; *CEP290*: centrosomal protein 290; ABCA4: ATP-binding cassette subfamily 4; XLRS: X-linked Retinoschisis; ATF6: Activating Transcription Factor 6; rd1: retinal degeneration 1; hiPSCs: Human-Induced Pluripotent Stem Cells; [4,6,16,67,68,69,71,72,83,85,96,97,102,104,105,106,107,108,109,110,111,112,113].

## 7. Advantages and Limitations of Organoids as Models for Studying Retinal Neurodegeneration

Retinal organoids represent substantial progress in mimicking retinal neurodegenerative disorders. They enable the real-time monitoring of processes such as apoptosis and autophagy and provide insights into ciliopathies at both the cellular and molecular levels. These organoids can be genetically manipulated and exposed to various experimental conditions, providing a versatile platform for therapeutic development. Among the advantages of organoids, it is important to highlight their ability to reproduce the retina’s three-dimensional architecture and differentiate into specific cell types, such as photoreceptors and RPE [19,23,24,25,26]. Traditional approaches included the in vitro culture of 2D human cell lines, explants, and animal models. One of the classic models for studying retinal degeneration is the rd10 mouse, commonly used as a model for RP [13]. However, it has been reported that up to 90% of clinical trials fail in the early stages, primarily due to the lack of reproducibility of results [14,48]. Another significant example lies in mutations in the *EYS* gene, one of the primary causes of autosomal recessive retinitis pigmentosa; however, there is no suitable model to study this disease, as EYS is not conserved in murine models. The existing data come primarily from other vertebrate models, such as zebrafish and Drosophila. In these cases, the development of human retinal organoids is crucial for a better understanding of the pathophysiology and for exploring new therapeutic strategies [114].

In this way, there is a lot of research in developing and obtaining retinal organoids from hiPSC or other somatic cells from patients who are carriers of gene mutations related to retinal degeneration. However, this type of model still has limitations compared to the human retina in vivo [5,12,23,95,115]. A great complication is the incomplete formation of outer-segment (OS) disks, crucial components for photoreceptor function, and one of the molecular and structural features of advanced photoreceptor differentiation [23]. These disks, essential for phototransduction, are house light-sensitive opsins conjugated with the chromophore 11-*cis*-retinal. Creating an appropriately stacked OS is one of the most difficult aspects of using retinal organoid technology; although mature retinal organoids exhibit OS-like structures loaded with opsins, they lack proper disk stacking and orientation [5,23].

Another problem is the interaction between NR cells and RPE cells, which provide metabolic support to the photoreceptors and allow nutrients to flow from the choroid. The RPE also contributes to the visual cycle by creating ionic gradients, converting all-trans-retinal to 11-cis-retinal, and secreting components for photoreceptor development, additionally participating in the phagocytosis of the OS of photoreceptors. In retinal organoids, RPE cells are juxtaposed to photoreceptors, partially mimicking the in vivo structure, but they often lack a complete monolayer [5,7,18,20,23,116]. A study of co-culturing RPE cells with retinal organoids in a microfluidic chip has been reported to increase the interactions with neighboring tissues. The RPE cells were plated under the retinal organoid, attaching to the photoreceptor OS, and phagocytic uptake of OS was observed in this system [117]. The drawback of this method is the difficulty of creating an in vitro retina that comprises all the neuronal and non-neuronal cells found in the human retina, including microglia and blood vessels [34]. As a result, the absence of a circulatory system during retinal organoid development causes hypoxia and starvation in inner cells such as RGCs [116]. Furthermore, the optic nerve fails to develop, causing RGC axons to stay within the organoid cavity and grow further, resulting in RGC loss [118]. The incorporation of these cells into retinal organoids would most likely require an assembled-type technique that assembles cells from many developmental lineages. For instance, an on-chip outer blood–retinal barrier model that combines endothelial and RPE cells in a microfluidic chamber has been reported [119].

In the center of the macula, the central fovea is populated by long- (L) and medium (M)-wavelength cones responsible for high-acuity vision. No macular structure is generated in retinal organoids [19,23,33]; however, the culture with factors such as the thyroid hormone [18] might be an inducer in cone-subtype specification. This absence restricts the modeling of diseases such as macular degeneration [5].

At the technical level, one of the main limitations of this technology resides in the complexity of the model and the long culture time, which does not allow the automation of the organoid production and differentiation process [116]. On the other hand, the process requires manual labor, which is inefficient in time when performing high-throughput drug screening experiments since many organoids are required [116]. Furthermore, to produce light-responsive lamellar retinal organoids in a format that allows for scalability and automation, the differentiation process highly depends on the nutrient availability. For example, it is essential to determine the ideal application of important growth factors and small molecules in addition to the cell seeding density [19,116]. All these elements increase capital consumption, the cost of reagents for stem cell-based cultures is high, and intensive manual labor is involved in certain steps of the differentiation protocol [115].

Another of the main challenges of this technology is the growth of different cell types in the same batch, generating great heterogeneity in size, a proportion of the different cell types, and stages of development [116]. In addition, the difference between iPSC cell lines and their capacity to generate organoids [26] must be considered, as well as the epigenetic control of the pluripotency of iPSCs [120]. These studies suggest that the epigenetic background, gene expression heterogeneity, and signaling activities of iPSCs all play an important role in the success of retinal organoid induction [26,34,120]. In addition, there is the challenge of characterizing models from hiPSCs with genetic mutations, the resulting phenotype of which is not known, causing quantitative changes that are difficult to relate to the results of the genetic mutation or are inherent variations between cell groups. Therefore, isogenic controls must be generated to differentiate the variations between diseased phenotypes and controls [116]. Finally, since normal retinal development continues beyond conception, retinal organoids do not provide this perspective on retinal diseases, especially in age-related disorders that require additional stressors to replicate in vivo phenotypes [57].

## 8. Future Perspectives and Challenges

In recent years, breakthroughs in retinal organoid technology have enabled the development of in vitro retinal neurodegenerative disease models; however, this technology is still in its early stages, with a gap compared to the in vivo retina. Consequently, future challenges are focused on the development of organoids with greater structural and functional complexity, the integration of the vascular network, and the development of an RPE bilayer [4,5,34,116].

Technically speaking, the obstacles are significant, as replicating a retina in vitro is extremely difficult due to its small dimensions, cell heterogeneity, and complex design [4,5]. One of the most pressing issues is the standardization of processes to improve efficiency and repeatability in organoid manufacturing, while also reducing the production time. This requires an understanding of the differences between iPSC cell lines and their ability to create organoids [26,34,120]. Current approaches for treating retinal organoids are time-consuming and, in most cases, require manual tissue separation or selection, which increases costs and impedes automation.

Furthermore, culture conditions must mimic physiological circumstances to promote retinal assembly, such as merging separately created cultures and tissues to improve crosstalk and using biomaterials for cell support. Advanced culture systems, such as biomaterials and scaffolds, are employed for this purpose. Bioreactors and robotic technologies for medium exchange, reagent addition, and organoid sorting and culture can boost the automation and scalability [115]. Similarly, the use of bioreactors to improve aeration and organ-on-a-chip approaches to incorporate vasculature should be considered to improve differentiation and maturation, whereas the co-culture of brain and retinal organoids (assembled technology) can generate appropriate NR-brain connections [18,50,117,119].

Another notable challenge is the heterogeneity of organoid cultures. This heterogeneity arises from the multi-ocular differentiation process, with some organoids developing certain regions more extensively than others [41]. For example, some organoids exhibit more RPE, others develop more NR, and others display a more prominent corneal area (Figure 2). Due to the wide variety of cell types present, transcriptomic studies are not highly recommended in this context. Instead, single-cell studies should be employed to obtain more precise results [33].

A functional cell investigation is also required. Therefore, retinal organoid technology facilitates both omics studies and the morphological and molecular characterization of these models for in vitro studies. As a result, a computational analysis of retinal organoid transcriptome data is required since it offers information on new aspects of cell growth and can help validate differentiation techniques. Moreover, downstream proteome and metabolomic studies inform proper functioning [18,33].

While retinal organoids have demonstrated great potential, several challenges remain to be addressed for their clinical application and use in translational research. Improving organoid maturation, enhancing vascularization, and integrating more complex functional studies are key areas for future investigation.

## 9. Conclusions

Due to the great clinical and genetic heterogeneity of RDDs and the limited knowledge of the common signaling pathways that cause the death of these cells, it is necessary to generate study models from the cells of patients with this type of disease. Advances in the generation, differentiation, and production of retinal organoids derived from hiPSCs provide new opportunities to elucidate the molecular and pathophysiological mechanisms involved in neurodegenerative diseases. Likewise, this new technology bridges the gap between in vitro and in vivo models, holding high potential for the application of personalized medicine as well as in the screening of new drugs and therapies. However, this technology still has several limitations in terms of time, cost, and variability, posing challenges for its widespread use in basic and translational research. In this regard, combining retinal organoids with emerging technologies such as 3D bioprinting could be a promising approach to achieving more homogeneous and reproducible cultures, further enhancing their applicability in research and therapeutic development.

## Figures and Tables

**Table 2:** Retinal Organoids Models for the study of pathophysiology of IRDs [4,6,67,68,69,71,72].

Disease	Gene	Studies on iPSC Lines’ Retinal Organoids with Induced or Naïve Disease-Specific Mutations	Reference	
Stargardt disease	*ABCA4*	Mutation expression	Su et al. (2022)	[77]
LCA	*CEP290*	Retinogenesis, morphology, markers, and pathogenic processes	Parfitt et al. (2016)	[78]
			Dulla et al. (2018)	[79]
			Shimada et al. (2017)	[80]
	*AIPL1 (LCA 4)*	Study of expression, therapy with the PTC124 drug	Lukovic et al. (2020)	[81]
	*AIPL1*	Mutation expression	Leung et al. (2022)	[82]
	*CRX*	Development and opsin expression	Chirco et al. (2021)	[83]
			Kruczek et al. (2021)	[84]
JSRD	*CEP290*	Retinogenesis, morphology, markers, and pathogenic processes	Shimada et al. (2017)	[80]
RP	*CRB1*	Compound heterozygous mutations	Boon et al. (2023)	[85]
	*PRPF31*	Mutation expression	Buskin et al. (2018)	[50]
	*USH2A*	Mutation expression	Guo et al. (2019)	[86]
	*TBC1D32*	Mutation expression	Xu et al. (2024)	[87]
Late-onset RP	*PDE6B*	Mutation expression	Bocquet et al. (2023)	[88]
X-linked RP	*RPGR* (ORF15 region, and intron 11)	Mutation expression	McDonald et al. (2024)	[69]
	*RP2*	Mutation expression	Lane et al. (2020)	[89]
	*RPGR*	Knockout animal models’ mutation	Lane et al. (2020)	[89]
Non-syndromic RP	*USH2A*	Mutation expression	Guo et al. (2019)	[86]
Usher syndrome type 1	*USH1B—MYO7A*	Mutation expression	Leong et al. (2022)	[90]
Usher syndrome type 2	*USH2A*	Mutation expression	Guo et al. (2019)	[86]
			Sanjurjo-Soriano et al. (2023)	[91]
Autosomal dominant optic atrophy	*OPA1*	Mutation expression	Lei et al. (2024)	[92]
XLRS	*RS1*	Mutation expression	Duan et al. (2024)	[93]
		Retinal development and expression of retinoschisin	Huang et al. (2019)	[94]
Retinoblastoma	*RB1*	Cell-to-cell interactions	Xu et al. (2024)	[87]

*ABCA4*: ATP-binding cassette subfamily 4; LCA: Leber Congenital Amaurosis; *CEP290*: centrosomal protein 290; *LCA* 4, 5, and 10: Leber Congenital Amaurosis types 4, 5, and 10; *AIPL1*: Aryl Hydrocarbon Receptor Interacting Protein-like 1; *PTC124*: Ataluren; *CRX*: Cone–rod homeobox; *JSRD*: Joubert Syndrome and Related Disorders; *CRB1*: Crumbs homolog 1; *PRPF31*: Pre-mRNA Processing Factor 31; *USH2A* and 1B: Usher syndrome type 2A and 1B, respectively; *OPA1*: Optic Atrophy 1; *ORF15*: Open Reading Frame 15; *RP2*: Retinitis Pigmentosa 2; *MYO7A*: myosin VIIA; *RS1*: Retinoschisin 1; *RB1*: Retinoblastoma 1.

## Abbreviations

AAV	Recombinant Adeno-Associated Virus
AAV2	AAV serotype 2
AAV5	AAV serotype 5
AAV2-7m8	AAV serotype 2-7m8
ABCA4	ATP-binding cassette subfamily 4
AIPL1	Aryl Hydrocarbon Receptor Interacting Protein-like 1
ASCs	Adult Stem Cells
ATF6	Activating Transcription Factor 6
AMD	Age-Related Macular Degeneration
Cas9	CRISPR-associated protein 9
CEP290	Centrosomal protein 290
CRB	Crumbs homolog
CRB1	Crumbs homolog 1
CRISPR	Clustered Regularly Interspaced Short Palindromic Repeats
CRX	Cone–rod homeobox
ECM	Extracellular Matrix
ESCs	Embryonic Stem Cells
EBs	Embryoid Bodies
hESCs	Human Embryonic Stem Cells
hiPSCs	Human-Induced Pluripotent Stem Cells
HDAC6	Histone Deacetylase 6
HIFs	Hypoxia-inducible factors
INL	Inner Nuclear Layer
IPL	Inner Plexiform Layer
iPSCs	Induced Pluripotent Stem Cells
IRD	Inherited Retinal Diseases
JSRD	Joubert Syndrome and Related Disorders
LCA	Leber Congenital Amaurosi
mESCs	Mouse Embryonic Stem Cells
mRNA	Messenger RNA
MYO7A	Myosin VIIA
NR	Neural retina
NPHP5	Nephrocystin-5
OFD1	Oral–Facial–Digital Syndrome 1
ONL	Outer Nuclear Layer
OPA1	Optic Atrophy 1
OPL	Outer Plexiform Layer
ORF15	Open Reading Frame 15
OS	Outer Segment
OV	Optic Vesicle-like
PRGR	Retinitis Pigmentosa GTPase regulator
PRPF31	Pre-mRNA Processing Factor 31
PTC124	Ataluren
QR110	Sophora RNA oligonucleotide therapeutic designed to correct the splicing defect in the CEP290
RB1	Retinoblastoma 1
RDD	Retinal degeneration diseases
rd1	Retinal degeneration 1
RGCs	Retinal Ganglion Cells
RPGRORF15	Isoform of the RPGR gene
RNA	Ribonucleic Acid
RP	Retinitis Pigmentosa
RP2	Retinitis Pigmentosa 2
RPE	Retinal pigment epithelium
RS1	Retinoschisin 1
USH2A	Usher syndrome type 2A
USH1B	Usher syndrome type 1B
WT	Wild-Type
XLRS	X-linked Retinoschisis
3D	Three dimensions(al)
